# 

**Published:** 1889-10

**Authors:** 


### CONTENTS—OCTOBER, 1889—VOL. 5, NO. 4.

ORIGINAL COMMUNICATIONS— Page.

Some Observations on the Possibilities of Preventive Surgery. By E. J. Ward, A. M., M. D. . . . . . . 119

Multiple Colloid-Cystoma of Left Ovary Successfully Removed. By R. Menger, M. D. . . . . . . . . . 132

A Portable Gynecological Table. By L. H. Luce, M. D. 138

SOCIETY NOTES—

Austin District Medical Society, Southern Surgical and Gynecological Association . . . . . . . . . . . . 144

EDITORIAL—

Dr. F. T. Paine's Discoveries in Electro-Therapeutics . 145

Texas as a Health Resort . . . . . . . . . . . . . . 147

Where's the Melancholy ? . . . . . . . . . . . . . . 149

Biography Contemporary Physicians of Texas . . . . 144

BOOK NOTICES AND REVIEWS—

Transactions Texas State Medical Association, 1889. . 150

Cyclopœdia Diseases of Children; Keating; Vol. II. . 152

American Armamentarium Chirurgicum; Tieman . . 153

Favorite Prescriptions of Distinguished Practitioners . 153

Text Book of Medical Jurisprudence, etc. . . . . . . 154

MEDICAL NEWS AND MISCELLANY—

Numerous Items of News of Interest to Doctors . . . 154

False Swearing. Letter from Tarrant & Co . . . . . 156

PUBLISHER'S NOTES—

In which are many Kernels worth Picking out . . . . 159

